# SuPreme Study: a protocol to study the neuroprotective potential of sulfate among very/extremely preterm infants

**DOI:** 10.1136/bmjopen-2023-076130

**Published:** 2023-07-14

**Authors:** Elizabeth M Hurrion, Nadia Badawi, Roslyn N Boyd, Catherine Morgan, Kristen Gibbons, Stefanie Hennig, Pieter Koorts, Manbir Chauhan, Francis Bowling, Vicki Flenady, Sailesh Kumar, Paul A Dawson

**Affiliations:** 1 Department of Newborn Services, Mater Mothers' Hospital, Brisbane, Queensland, Australia; 2 Mater Research Institute The University of Queensland, South Brisbane, Queensland, Australia; 3 Cerebral Palsy Alliance, The University of Sydney, Sydney, New South Wales, Australia; 4 Queensland Cerebral Palsy and Rehabilitation Research Centre, The University of Queensland, Saint Lucia, Queensland, Australia; 5 Child Health Research Centre, Mater Research Institute The University of Queensland, South Brisbane, Queensland, Australia; 6 School of Clinical Sciences, Queensland University of Technology Faculty of Health, Kelvin Grove, Queensland, Australia; 7 Integrated Drug Development, Certara Strategic Consulting, Certara LP, Princeton, New Jersey, USA; 8 Grantley Stable Neonatal Unit, Royal Brisbane and Women's Hospital, Herston, Queensland, Australia; 9 Department of Newborn Care, Gold Coast University Hospital, Southport, Queensland, Australia; 10 Walter and Eliza Hall Institute of Medical Research, Melbourne, Victoria, Australia

**Keywords:** NEONATOLOGY, Developmental neurology & neurodisability, Paediatric neurology

## Abstract

**Introduction:**

Antenatal maternal magnesium sulfate (MgSO_4_) administration is a proven efficacious neuroprotective treatment reducing the risk of cerebral palsy (CP) among infants born preterm. Identification of the neuroprotective component with target plasma concentrations could lead to neonatal treatment with greater efficacy and accessibility.

**Methods and analysis:**

This is a prospective observational cohort study, in three tertiary Australian centres. Participants are preterm infants, irrespective of antenatal MgSO_4_ exposure, born in 2013–2020 at 24^+0^ to 31^+6^ weeks gestation, and followed up to 2 years corrected age (CA) (to September 2023). 1595 participants are required (allowing for 17% deaths/loss to follow-up) to detect a clinically significant reduction (30% relative risk reduction) in CP when sulfate concentration at 7 days of age is 1 SD above the mean.

A blood sample is collected on day 7 of age for plasma sulfate and magnesium measurement. In a subset of participants multiple blood and urine samples are collected for pharmacokinetic studies, between days 1–28, and in a further subset mother/infant blood is screened for genetic variants of sulfate transporter genes.

The primary outcome is CP. Surviving infants are assessed for high risk of CP at 12–14 weeks CA according to Prechtl’s Method to assess General Movements. Follow-up at 2 years CA includes assessments for CP, cognitive, language and motor development, and social/behavioural difficulties.

Multivariate analyses will examine the association between day 7 plasma sulfate/magnesium concentrations with adverse neurodevelopmental outcomes. A population pharmacokinetic model for sulfate in the preterm infant will be created using non-linear mixed-effects modelling.

**Ethics and dissemination:**

The study has been approved by Mater Misericordiae Ltd Human Research Ethics Committee (HREC/14/MHS/188). Results will be disseminated in peer-reviewed journal publications, and provided to the funding bodies. Using consumer input, a summary will be prepared for participants and consumer groups.

**STRENGTHS AND LIMITATIONS OF THIS STUDY**

- The SuPreme Study examines an entirely novel hypothesis—that the neuroprotective component of antenatal magnesium sulfate is sulfate—which has a strong scientific basis.
- This study is powered to detect a clinically significant reduction (30% relative risk reduction) in the proportion of infants with cerebral palsy (CP) when sulfate concentration at 7 days of age is 1 SD above the mean.
- An early marker of risk using the General Movements Assessment, and follow-up to 2 years corrected age ensures excellent case ascertainment of the primary outcome; CP.
- Multiple sulfate measurements over the first 28 days after birth in a significant subgroup of infants allows pharmacokinetic population modelling, which is critical to designing a neuroprotective sulfate supplementation trial if the hypothesis is proven.
- The main limitation of the SuPreme Study is the risk of loss to follow-up, with potential to reduce the study’s power.

## Introduction

More than 1 in 10 births are preterm (<37 weeks gestation),^1^ with survivors at considerable risk of life-long disabilities, including cerebral palsy (CP), intellectual impairment, social/behavioural difficulties and educational disadvantage.^2 3^ The family, health and broader societal costs are estimated at >$26 billion per annum in the USA alone.^4^

CP is a permanent condition of motor dysfunction, is the most common physical disability in childhood, and almost 50% of cases are due to preterm birth.^5^ CP has a higher disability burden than blindness, deafness, severe asthma or diabetes.^6^ Thus, any intervention that reduces the risk of CP among preterm infants, particularly a simple, low-risk, low-cost treatment, would have considerable personal and economic benefits.

Antenatal maternal magnesium sulfate (MgSO_4_) therapy is established as neuroprotective for preterm infants,^7 8^ and is now standard of care in Australia for women at risk of imminent preterm birth <30 weeks gestation.^8^ Meta-analysis of high-quality randomised controlled trials has demonstrated a 29% risk reduction in the rate of CP, and a 40% reduction in substantial gross motor dysfunction for preterm infants exposed to MgSO_4_ before birth.^7^ However, approximately 40% of mothers of preterm infants born <32 weeks gestation in Australia and New Zealand do not receive this treatment, often due to ineligibility or urgent birth.^9^ If the precise neuroprotective component of MgSO_4_ can be identified, there is potential for direct treatment of preterm newborns, with universal accessibility, to prevent neurological sequelae.

Magnesium is the putative neuroprotective component, yet there is little supportive evidence for this. Specifically, there is no evidence to suggest that magnesium deficiency or altered pharmacodynamics are present in preterm infants that antenatal MgSO_4_ could correct or overcome.^10–12^ Furthermore, studies of preterm infants have shown no association between serum Mg concentrations (at 24–48 hours of age) and motor impairment including CP^12^ and some evidence of harm from high Mg concentrations at birth which have been associated with lower locomotor scores in infancy.^13^ Maternal MgSO_4_ treatment is not without risk as there is potential for magnesium toxicity, with life-threatening cardiac and respiratory effects. Because of this, women on MgSO_4_ treatment require close monitoring,^8^ which is one of the economic limitations preventing universal use, particularly in resource-poor countries.

### Role of sulfate in fetal brain development

Sulfate is an obligate nutrient for fetal growth and development,^14–16^ and the fetus is reliant on maternally supplied sulfate via active transport across the placenta.^17^ During the embryonic period of brain development, heparan sulfate proteoglycans (HSPGs) play crucial roles in neurogenesis, cell migration, axon guidance, synaptogenesis and plasticity, with cell signalling controlled by sulfonation patterns of the heparan sulfate chains.^18 19^ Sulfate is also critical to white matter structure and function; sulfatide (cerebroside sulfate) forms a structural component of the myelin sheath, and is critical to its insulative properties.^20^ As CP arises from white matter injury, these functions are particularly pertinent.

A sulfate-specific transporter, SLC13A4, is expressed in placental syncytiotrophoblasts.^21 22^ Targeted disruption of the *Slc13a4* gene in mice leads to fetal sulfate deficiency and severe developmental phenotypes that increase in severity as gestation progresses, including perturbed vessel formation and haemorrhage.^15 23^ Similarities to adverse neurodevelopmental outcomes and vascular abnormalities (intraventricular haemorrhage (IVH), retinopathy of prematurity (ROP) and capillary haemangiomas) among preterm infants suggest a potential origin in postnatal sulfate deficiency.

SLC13A4 is also expressed in the brain where it plays an important role in supplying sulfate for the sulfonation of HSPGs in the hippocampus. Targeted disruption of *Slc13a4* gene in mice reduces sulfate supply to the brain, leading to deficits in social interaction and memory in adulthood.^24^ Importantly, administration of a source of sulfate within a defined developmental window prevented these deficits.^24^ These deficits show similarities to adverse neurodevelopmental outcomes widely recognised among preterm infants.^2^

Maternal circulating sulfate concentrations increase more than twofold in pregnancy^25 26^ due to increased sulfate reabsorption in the maternal kidneys which is mediated by increased renal expression of the *SLC13A1* gene.^21 27^ Disruption of *SLC13A1* in humans and mice causes sulfate wasting and hyposulfataemia; mutations resulting in significant loss-of-function lead to reduced fetal survival and adverse neurodevelopment of offspring.^27–30^ The hyposulfataemic impact of the loss-of-function *SLC13A1* variant N174S (allelic frequency approximately 30%) will be greater if the mother is homozygous for the variant (8% of the population), and/or if the fetus has inherited the variant.^31^

### Sulfate deficiency in the neonatal period following very preterm birth

Infants born <32 weeks gestation (very preterm (VP)/extremely preterm (EP)) will likely rapidly become deficient in sulfate due to three factors: (1) minimal dietary supply of free inorganic sulfate in breast milk (including colostrum),^32^ total parenteral nutrition solutions, and human milk fortifiers; (2) negligible capacity to generate sulfate from sulfur-containing amino acids due to low expression of cysteine dioxygenase gene^14 33^; (3) immature renal function including low expression of SLC13A1, with limited ability to reabsorb filtered sulfate.^15 21 34 35^

Maturation of various physiological systems in the preterm infant accelerates postnatally, for example, surfactant protein production^36^ and renal maturation.^35^ Hence, it is likely that preterm infants rapidly switch on expression of the cysteine dioxygenase and SLC13A1 genes after birth, and thus develop the capacity to generate and retain sulfate within days to weeks.

A pilot study at the lead study site (online supplemental file 1) demonstrated that, as predicted, sulfate concentrations among VP/EP newborns fell rapidly over the first week after birth, before stabilising and increasing thereafter. As VP/EP infants are born during a critical period of brain development, they are likely to be highly vulnerable to adverse effects of hyposulfataemia. This window of vulnerability also coincides with the occurrence of IVH and ischaemia (later manifesting as periventricular leukomalacia (PVL)), both predisposing factors for adverse neurodevelopmental outcome. The pilot study at the lead study site (online supplemental file 1) also showed that antenatal MgSO_4_ largely mitigates the occurrence of very low concentrations in the preterm infant; boosting sulfate to supra-physiological concentrations at birth which then decay to normal or adequate concentrations by which time the infant’s capacity to generate and retain sulfate may have matured. However, common reduced-function variants of renal *SLC13A1* and placental *SLC13A4* sulfate transporter genes may impede the efficacy of antenatal MgSO_4_ treatment to boost fetal sulfate concentrations.

10.1136/bmjopen-2023-076130.supp1 Supplementary data

Given the strong scientific rationale for sulfate as the neuroprotective component of antenatal MgSO_4_, the aim of this study is to test the hypothesis that among VP/EP infants, severity of hyposulfataemia in the first week after birth correlates with adverse neurodevelopmental outcome. The primary outcome is CP, given the findings of the Cochrane meta-analysis of antenatal maternal MgSO_4_ treatment, but as per the recommendations of the Cochrane Review,^7^ other neurodevelopmental outcomes will be studied, including motor delay, cognitive function, language development and social/behavioural difficulties. This is particularly relevant given that animal studies suggest that sulfate deficiency has widespread effects on neurodevelopment, not confined to white matter abnormalities alone.^24 28–30 37^ Furthermore, vascular abnormalities which may be related to sulfate deficiency^15 23^ such as IVH, ROP, capillary haemangiomas will also be ascertained.

## Research hypothesis and aims

### Hypothesis

Among VP/EP preterm infants (<32 weeks gestation), plasma sulfate concentration at 7 days of age inversely correlates with adverse neurodevelopmental outcome.

### Primary aims

1. To determine whether there is a relationship between plasma sulfate and/or magnesium concentration in VP/EP infants at 7 days of age and formal diagnosis of CP at 2 years corrected age (CA), or absent fidgety movements^38^ at 12–14 weeks CA as a surrogate outcome when formal clinical assessment is unavailable, with subgroup analyses for EP and VP infants.
2. To determine whether there is a relationship between plasma sulfate and/or magnesium concentration in VP/EP infants at 7 days of age and any adverse neurodevelopmental outcome at 2 years CA; motor, cognitive and language scores as assessed by Bayley Scales of Infant and Toddler Development (Bayley III/4^A&NZ^),^39 40^ behavioural difficulties as assessed by Strengths and Difficulties Questionnaire (SDQ),^41 42^ and a composite ‘any moderate-severe disability’, with subgroup analyses for EP and VP infants.
3. To determine the optimal threshold of plasma sulfate and/or magnesium concentration identifying participants with risk of each adverse outcome; CP, motor/cognitive/language delay, behavioural difficulties, thereby identifying a potential plasma sulfate/magnesium target concentration for supplementation. Separate analyses will be performed for each adverse outcome, and for the composite ‘any moderate-severe disability’, along with separate analyses for EP and VP infants.

### Secondary aims

1. To explore changes in plasma sulfate concentrations over time, by correlating with the fractional excretion index (FEI) of sulfate at 1, 3, 7, 14 and 28 days of age, for VP/EP infants exposed to antenatal MgSO_4_ versus unexposed.
2. To explore whether, for VP/EP infants exposed to antenatal MgSO_4_, known loss-of-function variants in the maternal/infant *SLC13A1* or maternal *SLC13A4* genes are associated with lower plasma sulfate concentrations at 7 and 28 days of age.
3. To explore the impact of antenatal, neonatal and genetic factors (including MgSO_4_ dose, FEI sulfate and sulfate transporter gene variants) on neonatal sulfate concentrations within the first 28 days after birth, and to establish a population pharmacokinetic model for sulfate in the VP/EP infant.
4. To explore potential relationships between plasma sulfate concentration in VP/EP infants at 7 days of age and IVH (worst grade during neonatal intensive care unit (NICU) admission), ROP (worst stage in early infancy) or capillary haemangiomas (number and size in infancy).

## Methods

Prospective observational study at three tertiary NICUs in South-East Queensland, Australia: Mater Mothers’ Hospital (MMH)—lead study site, Royal Brisbane and Women’s Hospital (RBWH) and the Gold Coast University Hospital (GCUH).

### Participant cohort

#### Inclusion criteria

**Figure 1:**
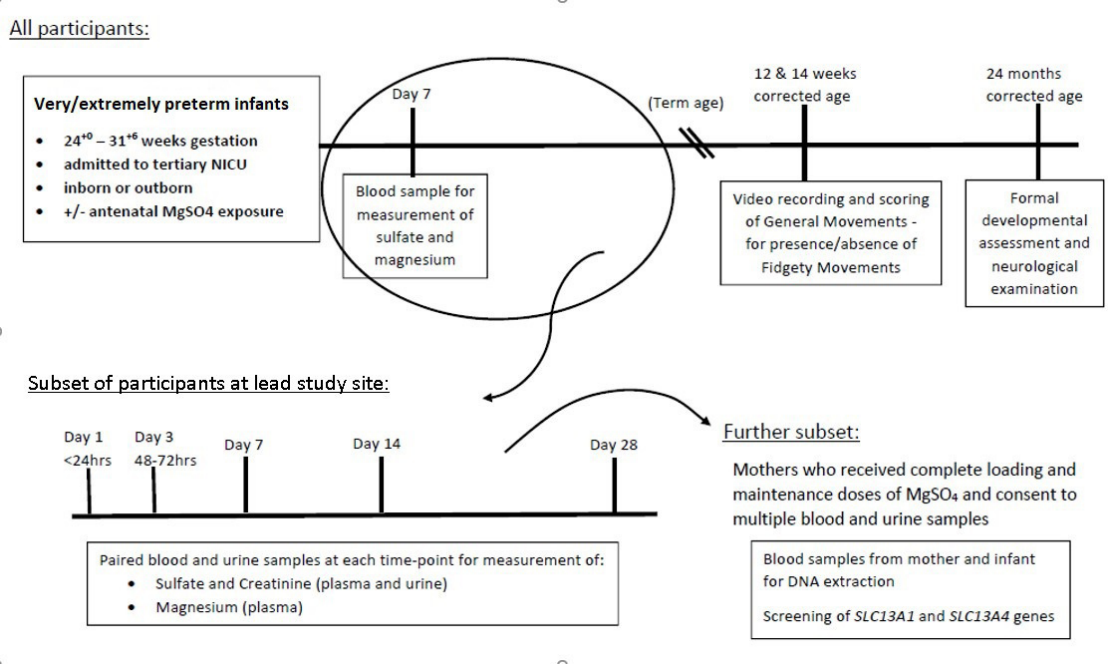
Protocol with details of biological sample collection. NICU, neonatal intensive care unit.

Preterm infants born at 24^+0^ to 31^+6^ weeks of gestation and admitted to NICU (figure 1).

#### Exclusion criteria

Major congenital/chromosomal abnormality likely to adversely affect survival or neurodevelopmental outcome. Maternal age less than 16 years.

### Recruitment

Women and their partners (‘parents’) are approached before or after delivery to explain the study, and consent is sought within the first few days after delivery.

Recruitment commenced at MMH in July 2015 and at RBWH in August 2015. To accelerate recruitment, the study was expanded to GCUH in June 2019.

Parents of surviving infants recruited into a pilot study of postnatal sulfate concentrations conducted at the lead study site in 2013–2014 (N=52) were contacted for consent to follow-up and inclusion in this study.

### Biological sample collection

#### Blood sample collection for *all* participants to address the primary aims

A blood sample (0.5 mL) is collected on day 7 of age (±1 day) for plasma sulfate and magnesium measurement (figure 1).

#### Additional biological sample collection for participant subsets to address the secondary aims

Parents at the lead study site (MMH) are approached to provide additional consent for multiple neonatal samples for pharmacokinetic studies (figure 1). A subset of these are offered the option to also consent to collection of maternal and infant blood samples for genetic studies (*SLC13A1* & *SLC13A4* gene variants). To be eligible for this subset, mothers had to have received complete loading and maintenance (at least 24 hours) doses of MgSO_4_ (figure 1).

Blood and urine samples are collected on days 3 (48–72 hours), 7, 14, 28 (figure 1). These days correspond to routine blood collections as per clinical guidelines at the lead study site. Urine samples are collected by placing a cotton wool ball in the nappy. When wet, the cotton ball is placed in a sterile container. Sulfate and creatinine are measured in urine and plasma samples for calculating the FEI sulfate. Magnesium is also measured in all plasma samples.

From October 2018 ‘day 1’ blood and urine samples are collected and stored on admission or within 24 hours of age for all NICU admissions 24–32 weeks gestation. The SuPreme Study information and consent process offers the option to consent to this ‘day 1’ sample being processed (figure 1). If consent is not obtained, the ‘day 1’ samples are discarded.

### Follow-up procedures

When participants are 12–14 weeks CA, parents record two videos of their infant at home via the BabyMoves app^43^ which provides a notification reminder when each video is due and uploads the videos for scoring (figure 1). Videos are scored independently by two expert assessors according to Prechtl’s method of qualitative assessment of general movements, fidgety period.^38^ Each assessor observes both videos of an infant and independently assigns one score; fidgety movements present (FM+)/fidgety movements absent (FM−)/abnormal fidgety movements. In the case of disagreement between the assessors, a third independent assessor reviews the videos. The assessors are not staff members at any of the recruitment centres, and are blinded to any clinical information, other than all infants being VP/EP and their CA when the video was taken. Infants assigned ‘FM−’ are identified as ‘high risk of CP’ and appropriate clinical referrals are made. If there is subsequently no further follow-up information regarding formal diagnosis of CP, these infants are classified as having CP.

Subsequent follow-up is arranged with the child’s main caregiver (biological mother in most cases).

Participants undergo a comprehensive face-to-face neurodevelopmental assessment at 2 years CA (figure 1).

A formal diagnosis of CP (or absence thereof) is established by clinical neurological examination by a paediatrician/neonatologist. If CP is diagnosed, the motor type, distribution and functional level on the Gross Motor Functional Ability Classification System (GMFCS) are recorded.^44 45^

Formal assessment of motor, cognitive and language outcomes is conducted using the Bayley Edition III/4^A&NZ^.^39 40^ Scores are calculated using CA.

Deafness and blindness are ascertained by caregiver report and paediatrician/neonatologist assessment, plus ophthalmology/audiology reports if available.

Social/behavioural outcomes are assessed through caregiver completion of the SDQ.^41 42^

If assessment at 2 years CA has not been attended, caregivers are invited to attend at any point thereafter. If the child is beyond the age range for the Bayley assessment, cognitive assessment is performed using the Wechsler Preschool and Primary Scale of Intelligence (WPPSI-IV^A&NZ^).^46^

Children enrolled in standardised routine neurodevelopmental follow-up (EP or birth weight below 1000 g) may also have a face-to-face assessment at 4 years CA using WPPSI-IV^A&NZ^ plus a neurological examination, and these data are also collected if available.

If face-to-face assessment is declined or not achieved, information is obtained by telephone interview with the child’s caregiver which includes questions on medical and developmental history, any CP diagnosis, and by standardised caregiver-completed developmental questionnaire (Ages and Stages Questionnaires (ASQ-3)^47^) and social/behavioural screening questionnaire (SDQ^41 42^). Telephone interviews are conducted by a psychology graduate who also has training and experience in telephone counselling, to optimise caregiver engagement. The ASQ-3^47^ is presented according to CA. CP is classified as ‘none’ if the caregiver reports that child was walking before 18 months CA and at the time of the telephone interview is running without difficulty and able to kick a ball with either foot. If the caregiver reports a diagnosis of CP, permission is sought to obtain further information from the child’s treating physician regarding CP diagnosis, motor type, distribution and GMFCS level. If the caregiver reports any motor difficulties, or if there are developmental concerns on the ASQ-3, the caregiver is encouraged to attend for face-to-face assessment, and if available these results are then used in place of ASQ-3 scores.

The COVID-19 pandemic resulted in temporary suspension of some follow-up services in 2020. As a result, follow-up data collection will continue until September 2023. This has delayed the age of formal follow-up assessment for some children, and some caregivers opted for telephone interview only, including ASQ-3 and SDQ.

### Biological sample processing

#### Sulfate and magnesium analysis of blood samples

All blood samples are processed at each site to isolate plasma which is then stored at −20°C. Plasma samples are transported to the lead study site where they are batch-analysed. Plasma sulfate concentrations are measured using ion chromatography with suppressed conductivity detection using a Dionex ICS2000, as previously described.^48^ Plasma magnesium concentrations are quantitated as per routine testing using a Vitros 5.1 FS chemistry analyser.

#### Analysis of plasma and urine samples to calculate FEI sulfate

Urine samples are stored at −20°C and batch-analysed. The cotton wool ball is thawed and centrifuged to obtain urine for analysis. Urinary and plasma sulfate concentrations are measured as previously described.^48^ Urinary and plasma creatinine is measured as per routine testing using a Vitros 5.1 FS chemistry analyser. FEI sulfate is calculated using the formula: (urinary sulfate mM×plasma creatinine mM)/(plasma sulfate mM×urinary creatinine mM).

#### Genetic analysis of DNA samples

DNA isolated from blood samples (QIASymphony, QIAGEN) of mother and infant pairs is screened for sequence variants in the *SLC13A4* gene (infant samples) and *SLC13A1* gene (maternal and infant samples) using paired end sequencing on a MiSeq sequencer (Illumina) running 2×150 bp chemistry version 2. Data analysis including alignment to the reference genome hg19 and variant calling is carried out using QIAGEN’s online GeneRead Variant Analysis portal.

### Antenatal and neonatal data

Details regarding dose/duration/timing of the loading dose and maintenance infusion of MgSO_4_, along with maternal weight, are recorded.

Antenatal/maternal and delivery data are also recorded, including antenatal steroid administration, primary reason for preterm birth, whether inborn or outborn from tertiary centre, mode of birth, and date and time of birth.

Neonatal data collected includes expected date of delivery, gestational age and weight at birth, sex, singleton/multiple, Apgar scores (1 and 5 min). CRIB score^49^ data are collected if available. Details of potential factors affecting plasma sulfate concentration are also noted, specifically date/time of any blood transfusions administered within the blood sampling period, and date when ferrous sulfate commenced.

### Short-term outcome data

#### IVH and PVL

Worst grade of IVH (0-IV) and worst PVL (including areas involved, laterality and presence of cystic changes) are coded from reports of routine cranial ultrasound scans on days 5 and 28, and at corrected GA 34–36.

#### Retinopathy of prematurity

Worst stage of ROP (0–5), for each eye, including presence of ‘plus’ disease, and any treatment interventions, is coded from routine ophthalmological review.

#### Capillary haemangioma

Number and size of any capillary haemangiomas is recorded prior to discharge, and by caregiver report at 12 weeks CA. This information is supplemented by caregiver report and medical examination at the 2-year assessment.

#### ‘High risk’ of CP at 12–14 weeks CA

Prechtl’s method of qualitative assessment of general movements is used to identify the absence of fidgety movements.^38^ Absent fidgety movements at this age confers a very high risk for CP at 2 years of age, with sensitivity of 97.6% and specificity of 95.7%.^50–52^

### Outcome data at 2 years CA

#### CP diagnosis

CP is defined according to the recommendations of the Surveillance of Cerebral Palsy in Europe network.^53^ Functional severity of movement disability is classified according to the Gross Motor Function Classification System (GMFCS), which describes five levels according to functional abilities in sitting and walking and the need for assistive devices (walkers or wheelchairs), where level 1 indicates minimal functional impairment.^44 45^ The GMFCS descriptors appropriate to the child’s CA are used.

#### Cognitive and general development

#### Bayley Scales of Infant and Toddler Development (Bayley-III and Bayley-4^A&NZ^)

The Bayley Scales is a developmental assessment for young children up to 42 months old, yielding standardised composite scores which have a mean of 100 and an SD of 15 for three domains (cognitive, language and motor).^39 40^ The third edition (Bayley-III)^39^ is norm-referenced against US children and was used in this study until the fourth edition, Australia and New Zealand standardised edition (Bayley-4^A&NZ^)^40^ was available (2020).

#### Wechsler Preschool and Primary Scale of Intelligence

The WPPSI-IV^A&NZ^ is a measure of cognitive development for preschoolers and young children between the ages of 2 years 6 months to 7 years 7 months.^46^ It yields a Full-Scale IQ (FSIQ) and several subscales; above the age of 4 years these are: verbal comprehension, visual spatial, fluid reasoning, working memory and processing speed.^46^

#### Ages and Stages Questionnaires

The ASQ-3 is a parent/caregiver-completed developmental screening tool for young children up to 66 months of age.^47^ There are 21 age-specific questionnaires with assessments in five developmental domains: communication, problem-solving, personal-social, gross motor, fine motor skills.^47^ The score for each domain is categorised according to normative data obtained on US children.^47^ Scores below the 1 SD cut-off at 24 months using the ASQ-2 have reasonable sensitivity (83.6%) in identifying neurodevelopmental delay.^54^

#### Other disabilities and difficulties

Deafness is defined as severe auditory deficit requiring hearing aids or cochlear implant.

Blindness is defined as a severe bilateral visual deficit affecting function.

Social/behavioural difficulties are assessed using the SDQ for 2–4 years old, which is a parent/caregiver-completed behavioural screening questionnaire.^41 42^ There are 25 questions giving scores in five domains: emotional symptoms, conduct problems, hyperactivity/inattention, peer relationship problems, prosocial behaviour.^41 42^

#### Composite outcome of ‘any moderate-severe disability’

This is defined as any of the following:

1. CP, GMFCS levels 3–5.
2. Moderate to severe global developmental delay—cognitive and language scores on formal developmental assessment (eg, Bayley) more than 2 SD below mean.
3. Moderate to severe intellectual impairment—FSIQ on formal assessment (eg, WPPSI) more than 2 SD below mean.
4. In the absence of formal assessment, report by paediatrician or child development service of moderate/severe disability, or ASQ-3 more than 2 SD below mean in at least four domains.
5. Registered as blind.
6. Deafness requiring hearing aids or cochlear implant.
7. Severe behavioural difficulties (SDQ total difficulties score ‘very high’).

## Sample size and statistical analysis

### Sample size

The study was powered for identifying the relationship between plasma sulfate and/or magnesium and the primary outcome, diagnosis of CP (primary aim 1). At the lead study site (MMH), based on audit of data, the CP rate for VP/EP survivors was 7%. The published risk reduction of antenatal MgSO_4_ on CP as per the Cochrane Review of this therapy is 29%.^7^ As such, to determine a clinically significant reduction in the proportion of infants with CP at 2 years CA (7%–5.25%, corresponding to a 30% relative reduction and an OR of 0.74 when sulfate concentration is increased by 1 SD above the mean), and assuming 80% power, a type I error of 0.05 and using plasma sulfate as a continuous predictor, 1324 infants are required. This calculation is based on using logistic regression as the primary analysis technique, and allows for a set of confounders to be included, including plasma magnesium concentration. Allowing for 17% participant loss, including deaths, withdrawals and lost to follow-up, a sample size of 1595 infants will be required.

A sample size calculation for primary aim 2 has not been performed, as the Cochrane Review^7^ was unable to demonstrate a risk reduction effect for intellectual deficits or developmental delay. Cognitive and language delay in VP infants is however much more prevalent than CP, and these outcome measures are continuous variables (standard scores). The sample size calculated for primary aim 1 will therefore be sufficient to detect a clinically significant risk reduction for cognitive/language outcomes if present. Primary aim 3 is predominantly exploratory, and as such a sample size calculation was not undertaken, and p values will not be presented.

### Sample size considerations for secondary aims

#### Secondary aim 1

Using data obtained in a pilot study undertaken at the lead study site (online supplemental file 1), 231 infants exposed to antenatal MgSO_4_ are required to produce a two-sided 95% CI (SD 154, CI width 40). Additionally, 160 infants *not* exposed to antenatal MgSO_4_ are required to produce a two-sided 95% CI (SD 32, width 10).

#### Secondary aim 2

The sample size is based on the combined allelic frequencies of the *SLC13A1* (0.26% p.Arg12Ter and 29.5% p.Asn174Ser) and *SLC13A4* (0.05% p.Ser139Valfs, 0.02% p.Leu72Serfs and 0.05% p.Phe309Cys) gene variants from the NCBI database, and data from a pilot study undertaken at the lead study site showing low neonatal blood sulfate concentrations at 7 days in 10/21 (48%) and at 28 days in 5/15 (33%) of infants whose mothers received MgSO_4_ (online supplemental file 1). Since this aim encompasses two time points, the type I error will be set at 0.025 rather than 0.05 (using Bonferroni’s correction). Additionally, assuming power of 90%, 30% of infants will have a gene variant and in this group 90% will have low sulfate, compared with 30% in the non-variant group, 60 infants and their mothers will need to be included in the study at 7 days of age. At 28 days of age, with the same parameters, however, if fewer infants with a gene variant (80%) have low sulfate and more infants without a gene variant (20%) have low sulfate, 35 infants are required. These infants will be a subset of secondary aim 3.

#### Secondary aim 3

Using data obtained in a pilot study undertaken at the lead study site (online supplemental file 1), 121 infants exposed to antenatal MgSO_4_ are required to develop a multivariate model to assess the relationship between low plasma sulfate concentration with the dose of MgSO_4_ administered, FEI sulfate in the first week after birth, sampling time-point and absence or presence of known loss of function mutations, using the assumption that ten infants with low sulfate are required for potential independent variables.

Blood and urine samples from 300 infants (with or without antenatal MgSO_4_ exposure) taken within 24 hours after delivery, and at 3, 7, 14, 28 days of age will provide sufficient data to create and fit a population pharmacokinetic model for sulfate in the preterm infant using non-linear mixed-effects modelling.^55 56^

#### Secondary aim 4

A sample size calculation for secondary aim 4 has not been performed, as there is no information regarding a relationship between antenatal MgSO_4_ and IVH/ROP/haemangiomas. Results presented will be noted as exploratory.

### Statistical methods

#### Primary aims 1 and 2

Descriptive statistics will be used to present the antenatal, intra partum, post partum, neonatal and follow-up characteristics, including plasma sulfate and magnesium concentration, of the cohort. Bivariate relationships will be explored between these characteristics and CP and adverse motor/cognitive/language/behavioural outcomes. Mixed-effects regression models will examine the association between plasma sulfate and magnesium concentrations at 7 days of age with adverse outcomes; site will be included as a random effect, with characteristics known to be associated with the outcome from literature and clinical experience, as well as demonstrating an association in bivariate analyses, included as fixed effects. The number of factors included will be restricted dependent on the prevalence of the outcome. Both unadjusted and adjusted effect estimates will be presented, along with 95% CIs, and p values for the primary model examining CP. An a priori subgroup analysis for <28 weeks versus ≥28 weeks will be undertaken.

#### Primary aim 3

Receiver operator characteristic (ROC) curves will be constructed, using CP/motor delay/cognitive delay/language delay/behavioural difficulties as individual outcomes, and the sulfate concentration as the predictor. The area under the ROC curve (AUROC) will be calculated and presented, along with the 95% CIs. The sulfate concentration that results in the best combination of sensitivity and specificity will be calculated (Youden’s index) for each outcome.

#### Secondary aims

Multivariable analysis techniques will be used to address secondary aim 1 examining the association between plasma sulfate levels and FEI sulfate according to antenatal MgSO_4_ exposure, and secondary aim 2 examining the association between plasma sulfate levels and loss of function gene variants. Similarly, multivariate analyses will be used to address secondary aim 3, examining the association between plasma sulfate levels and FEI sulfate, maternal MgSO_4_ dose, and loss-of-function *SLC13A1* and *SLC13A4* gene variants. To further address secondary aim 3, non-linear mixed-effects modelling for repeated measures will be used to explore the sulfate concentrations-time turn-over profile in the preterm infant. Finally, multivariate analyses will be used to examine the association between plasma sulfate concentration at 7 days of age with IVH, ROP or capillary haemangiomas.

## Patient and public involvement

The strategic purpose of this study (prevention of CP and other disabilities) aligns with one of the four goals—prevention and cure—identified in the Australian and New Zealand Cerebral Palsy Strategy,^57^ developed collaboratively by people with CP, their families, practitioners and organisations involved in supporting people with CP.

A consumer advocate was involved in the original study design and draft protocol (in 2014), including considerations to guide acceptability of blood sampling and limiting the burden of follow-up procedures. The study design was adjusted according to this input. The consumer advocate was also involved in reviewing the draft parent information and consent form. They were not involved in recruitment or conduct of the study. The consumer is a parent of a preterm-born child with CP, who is familiar with the standard clinical follow-up processes on which this assessment protocol is based.

One of the authors, Badawi N, maintains a strong focus on consumer perspectives and advocacy through her role with CP Alliance, the foremost Australian organisation for CP, a consumer-led charity for people with CP and their families/carers.

In recognition of the benefits of, and increased expectations for patient and public involvement, greater consumer involvement is planned for the dissemination of results to participant families and consumer groups/media releases. This will include the original consumer advocate, parent support groups for preterm infants and CP Alliance (through Badawi N). This will occur as soon as any manuscript is accepted for publication, and will occur by focus groups to identify which results/conclusions are important and how they should be presented, followed by review of a draft participant newsletter and consumer group/media releases by individual consumers.

## Ethics and dissemination

The study has received ethics approval (HREC/14/MHS/188) from the Mater Misericordiae Ltd Human Research Ethics Committee (EC00332). Informed consent was given by parents of all participants before taking part.

Dissemination of results will initially focus on the primary aim. Results will be published in a peer-reviewed journal, presented at conferences and made available to the funding bodies. A lay summary will be developed using consumer input, and disseminated contemporaneously to participants (via an emailed newsletter), and local and national consumer groups (preterm birth and CP Alliance), as well as media releases if appropriate. Further results regarding secondary aims will then be published.

## Supplementary Material

Reviewer comments

Author's manuscript
