# Supplementary material for: SuPreme Study: a protocol to study the neuroprotective potential of sulfate among very/extremely preterm infants
Source: BMJ Open. 2023 Jul 14;13(7):e076130. doi: 10.1136/bmjopen-2023-076130 (PMC10351292; doi:10.1136/bmjopen-2023-076130)
Supplement: Supplementary data [file bmjopen-2023-076130supp001.pdf]

SUPPLEMENTARY FILE 1
Pilot study data used to calculate sample size for Secondary Aims.

Laboratory data from a pilot study conducted at the lead study site (MMH) in 2013-14 in preterm infants born 25-29 weeks gestation. At the time of the sample size calculation for this study, laboratory data was available for 33 infants (pilot study size N=52 on completion).

Laboratory data included plasma sulfate concentrations and urine creatinine and sulfate concentrations. Time points in the pilot study were admission to the NICU (0) and ages 7 days, 28 days and 35 weeks corrected gestation (K35).

Mean and 95% confidence intervals were calculated for plasma sulfate concentration and fractional excretion index, for each time-point, according to antenatal $MgSO_4$ exposure (+/-).

Mean and 95% confidence intervals for plasma sulphate concentrations.

	$MgSO_4$	*Plasma Sulfate (μM)*			
Age	***+/-***	***Mean***	***SD***	***(n)***	***95% CI***
0	+	693	396	(24)	526-860
0	-	250	170	(9)	119-380
7d	+	221	154	(20)	149-294
7d	-	85	32	(5)	45-125
28d	+	235	106	(14)	174-297
28d	-	124	79	(6)	41-208
K35	+	316	101	(5)	191-443
K35	-	229	85	(4)	93-366

Mean and 95% confidence intervals of fractional excretion index (FEI) for sulfate.

	$MgSO_4$	*FEI Sulfate*			
Age	***+/-***	***Mean***	***SD***	***(n)***	***95% CI***
0	+	0.52	0.24	23	0.42-0.63
0	-	0.50	0.28	5	0.16-0.85
7d	+	0.31	0.13	11	0.22-0.39
7d	-	0.27	0.15	6	0.17-0.43
28d	+	0.24	0.11	11	0.17-0.32
28d	-	0.21	0.09	5	0.09-0.32
K35	+	0.26	0.09	4	0.10-0.41
K35	-	0.18	0.08	3	0.00-0.37
