# Supplementary material for: SuPreme Study: a protocol to study the neuroprotective potential of sulfate among very/extremely preterm infants
Source: BMJ Open. 2023 Jul 14;13(7):e076130. doi: 10.1136/bmjopen-2023-076130 (PMC10351292; doi:10.1136/bmjopen-2023-076130)
Supplement: Reviewer comments [file bmjopen-2023-076130.reviewer_comments.pdf]

PEER REVIEW HISTORY

BMJ Open publishes all reviews undertaken for accepted manuscripts. Reviewers are asked to complete a checklist review form (**http://bmjopen.bmj.com/site/about/resources/checklist.pdf**) and are provided with free text boxes to elaborate on their assessment. These free text comments are reproduced below.

ARTICLE DETAILS

**TITLE (PROVISIONAL)**	SuPreme Study: A protocol to study the neuroprotective potential of sulfate among very/extremely preterm infants
**AUTHORS**	Hurrion, Elizabeth; Badawi, Nadia; Boyd, Roslyn; Morgan, Catherine; Gibbons, Kristen; Hennig, Stefanie; Koorts, Pieter; Chauhan, Manbir; Bowling, Francis; Flenady, Vicki; Kumar, Sailesh; Dawson, Paul

This article was not externally reviewed at BMJ Open. Protocols that have been independently assessed prior to submission to BMJ Open are usually fast-tracked to publication on the grounds that further substantial changes will not be possible. This independent assessment will usually be external, independent review for both a substantial grant award from a non-commercial or government funder and ethics approval.
